# The surgical management of a patient with Fuchs endothelial dystrophy and cataracts

**DOI:** 10.22336/rjo.2024.15

**Published:** 2024

**Authors:** Alina Gabriela Gheorghe, Ana Maria Arghirescu, Andrei Coleașă, Ancuța Georgiana Onofrei

**Affiliations:** * Department of Ophthalmology, Clinical Hospital for Ophthalmological Emergencies Bucharest, Bucharest, Romania

**Keywords:** DMEK, penetrating keratoplasty, Fuchs endothelial dystrophy, cataract surgery

## Abstract

**Objective:** To report the two different surgical approaches in the case of a patient with Fuchs endothelial dystrophy with low endothelial cell count and advanced cataracts.

**Methods:** The chosen surgical approach differed between eyes, with the right eye undergoing a combined approach consisting of cataract surgery, intraocular lens implantation, and penetrating keratoplasty in 2022. One year later, for the left eye, a different approach was decided: cataract surgery followed by Descemet membrane endothelial keratoplasty (DMEK). The Descemet membrane graft was prepared by the surgeon using the liquid bubble technique. AS-OCT was used to monitor the patient before and after surgery.

**Results:** Visual recovery was excellent for both eyes, however, visual acuity improved quickly in the left eye (DMEK), while, in the right eye (PK), the best corrected visual acuity was reached after several months post-surgery.

**Conclusion:** Advanced stages of Fuchs dystrophy patients will most likely need corneal transplantation. Each type of corneal transplantation procedure comes with unique challenges, both intraoperative and postoperative. DMEK is a very good treatment option for patients with Fuchs endothelial dystrophy, with excellent visual recovery and good graft survival at the 10-year mark.

**Abbreviations:** DMEK = Descemet membrane endothelial keratoplasty, PK = penetrating keratoplasty, AS-OCT = anterior segment optical coherence tomography, FECD = Fuchs endothelial corneal dystrophy, BCVA = best corrected visual acuity, US = ultrasound, CDE = cumulative dissipated energy, IOL = intraocular lens

## Introduction

A corneal dystrophy affecting the endothelium and Descemet membrane, Fuchs endothelial dystrophy (FECD) is more common in women. It usually presents with bilateral, asymmetric evolution, as in this case, in which the right eye was more affected than the left. Symptoms typically appear in the fifth-sixth decade of life. The first area to be affected is usually the central cornea. Patients frequently complain of seeing halos around lights and loss of contrast sensitivity, and with disease progression, visual manifestations worsen. Most patients maintain a functional visual acuity throughout life, with a significant improvement after cataract surgery. On the other hand, a small number of FECD patients, in whom the cell dysfunction is more advanced, will need a type of corneal transplantation to ensure the best visual recovery possible.

The availability of different surgical techniques for patients with corneal dysfunction has greatly improved patients’ chances for a better visual outcome. In the last ten years, lamellar keratoplasty techniques have been improved and standardized, therefore resulting in more favorable outcomes. Throughout the world, lamellar keratoplasty surgery is slowly replacing full-thickness keratoplasty as the most frequently performed procedure in FECD patients [**1**]. High-accuracy imaging of the cornea (AS-OCT) gives the surgeon a greater insight into the extent of the corneal dysfunction, therefore aiding the surgeon in deciding which type of keratoplasty is best suited for each case.

The times for visual rehabilitation differ significantly between the two types of keratoplasty presented in this manuscript. Each type of corneal transplant procedure comes with a series of intraoperative and postoperative challenges, both technique-wise and recovery-wise. As FECD patients typically also present with cataracts, the intraocular lens must be chosen per the type of keratoplasty that will be performed.

## Case report

The chief complaints were as follows: progressive visual acuity loss, diminishing contrast sensitivity, blurry contours, and halos around lights. The last three phenomena appeared approximately 15 years before, creating significant visual discomfort. 

A 71-year-old woman presented in our clinic in 2021 for progressive bilateral decrease in visual acuity, and was diagnosed with Fuchs endothelial dystrophy and senile cataracts. No other systemic diseases were reported. BCVA was counting fingers at 3 meters for the right eye, and 0.3 for the left eye. Intraocular pressure was within normal limits in both eyes. Findings at the slit lamp examination were as follows: asymmetrical evolution of the FECD, with the right eye being the most affected, with central corneal haze, Descemet folds, epithelial edema, and subepithelial bullae. The left eye was found with a beaten bronze aspect of endothelium and slightly elevated central corneal thickness, but no significant corneal haze or epithelial edema at this point. The lens in both eyes presented corticonuclear opacities. Endothelial cell density could not be evaluated in either eye. 

**Fig. 1:**
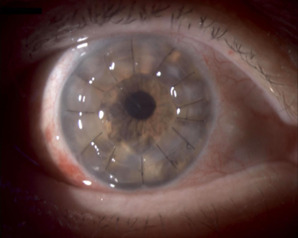
Right eye - penetrating keratoplasty at 1-month mark (personal photo archive - Dr. Alina Gheorghe)

In early 2022, given the significant decrease in visual acuity, a result of both the progression of the cataract, and the loss of corneal transparency due to endothelial cell dysfunction, a combined approach was chosen: phacoemulsification with monofocal intraocular lens implantation and penetrating keratoplasty. Visual acuity in this eye progressively increased after surgery: 0.2 BCVA one month after, 0.3 BCVA at the 3-month mark, and 0.9 BCVA at the 1-year mark. The graft maintained its centration and transparency. Endothelial cell density one year post penetrating keratoplasty was 2964 cells/mm2, evaluated using specular microscopy. Central corneal thickness was within normal limits (516 µm). The patient reported some higher-order aberrations after surgery, most likely due to the corneal surface irregularities typical in penetrating keratoplasty patients (**Fig. 1**). 

During visual rehabilitation for the right eye, visual acuity in the left eye continued to deteriorate: the patient complained of a higher-order aberration increase and a significant decrease of vision in the first part of the day becoming more frequent. A sequential approach was decided upon in early 2023, with phacoemulsification and intraocular lens implantation preceding DMEK by two months. Special considerations regarding the choice of intraocular lens in patients who undergo DMEK surgery have also been taken. An intraocular lens with a refractive target of -1D was chosen to compensate for the expected hyperopic shift after the DMEK intervention.

**Fig. 2:**
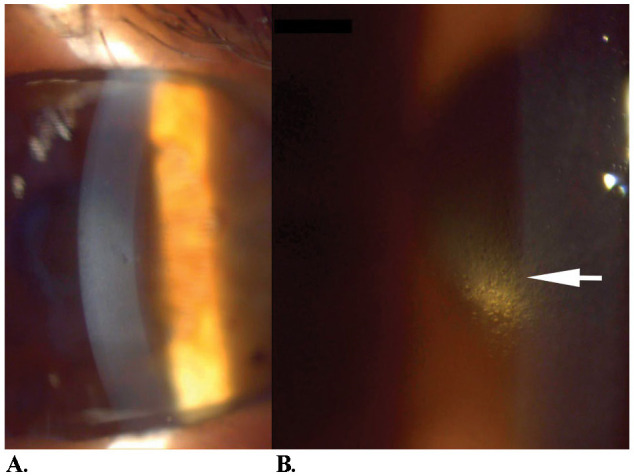
After cataract surgery, before DMEK. **A.** Corneal haze and subepithelial bullae; **B.** Beaten bronze aspect (personal photo archive of Dr. Alina Gheorghe)

Preoperatively, specular microscopy could not evaluate endothelial cell density. Central corneal thickness was also significantly above normal values, showing a significant endothelial cell dysfunction (674 µm). Controlled fluidics, with low US usage and low CDE, together with the soft-shell technique were used to ensure minimal endothelial cell loss. Despite the success of the cataract surgery, the improvement in visual acuity was not significant; biomicroscopy performed one month later showed further corneal decompensation with central corneal haze, epithelial bullae, and a more pronounced beaten bronze aspect centrally (**Fig. 2**). Descemet membrane endothelial keratoplasty (DMEK) was performed in April 2023. 

**Fig. 3:**
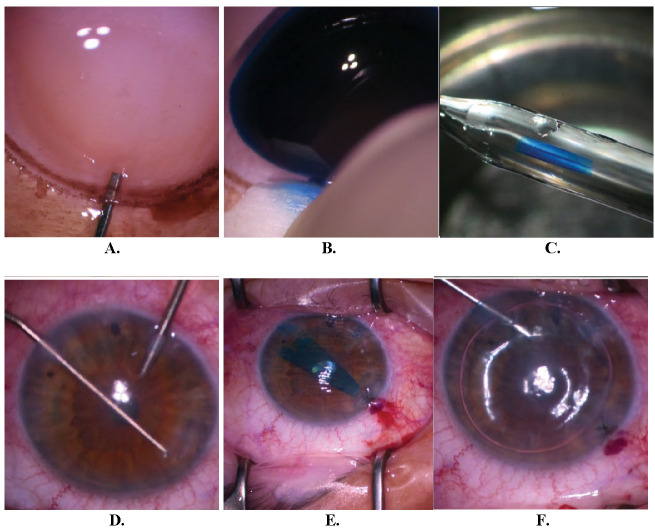
Endothelial graft preparation. **A.** Inserting the cannula in the predescemetic zone; **B.** Endothelial graft hydrodissection using trypan blue; **C.** Endothelial graft in double scroll configuration loaded in the glass cannula. Surgery: **D.** Descemetorhexis; **E.** Endothelial graft in double scroll configuration, inserted in the anterior chamber; **F.** Bubble placement after graft unscrolling and centration (personal photo archive of Dr. Alina Gheorghe)

**Fig. 4:**
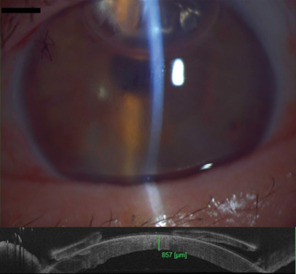
Above - one day after DMEK, the endothelial graft is attached, and the air bubble is still present in the anterior chamber; below - AS-OCT showing increased central corneal thickness one day after DMEK (personal photo archive of Dr. Alina Gheorghe)

**Fig. 5:**
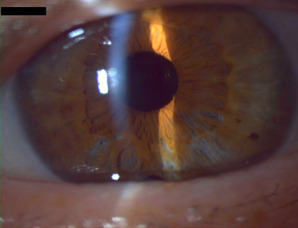
Left eye, 6 months post DMEK surgery (personal photo archive - Dr. Alina Gheorghe)

The surgeon preoperatively prepared the graft. Intraoperatively, good centration and graft attachment was achieved (**Fig. 3**). The patient was instructed to remain in a supine position 24 hours after surgery. One day postoperatively, graft attachment was confirmed using AS-OCT and slit lamp exam (**Fig. 4**). Central corneal thickness was evaluated at one day (857 µm), one month (579 µm), and six months (511 µm) post-DMEK. No rebubbling was done in this case. The endothelial graft remained attached (**Fig. 5**). 

One month after DMEK, visual acuity increased to 1 BCVA. The patient reported a significant decrease in higher-order aberrations. 

## Discussions

It is worth keeping in mind that, when evaluating a cataract patient with FECD, one should take note of both the age and the characteristics of the cataract. Periodic monitoring is necessary as the patient usually comes in complaining of loss of color contrast, and halos around lights. The moment of cataract surgery in such patients should be chosen wisely, given the increased risk of corneal complications, when compared to patients who do not suffer from FECD. An earlier intervention on a less advanced cataract might mean a lower surgical time, with lower CDE, therefore resulting in a better visual acuity post-surgery, delaying the need for corneal transplantation.

The surgeons must also consider the inevitable endothelial cell loss associated with cataract surgery so that they adapt their techniques to minimize it to the best of their ability, such as using a protective soft-shell technique, and sensible amounts of US during phacoemulsification [**2**]. A good understanding of fluidics is always an advantage for the surgeon.

AS-OCT and specular microscopy are valuable tools in evaluating the patient as they allow for a better risk assessment preoperatively. Preoperative central corneal thickness is a sensible predictor for corneal decompensation post-cataract surgery in FECD patients. Patients with central corneal thickness of more than 640 µm, together with an endothelial cell density of less than 1000 cells/mm2 are at a greater risk for corneal decompensation after cataract surgery [**3**]. 

For the patient who undergoes DMEK surgery, IOL calculations should be made considering, a target refraction of approximately -1D, to compensate for the expected hyperopic shift post-DMEK [**4**]. In penetrating keratoplasty patients, postoperative refractive error is difficult to predict.

Accurate preoperative Fuchs stage evaluation plays an important role in the choice of transplantation type, as well as the approach, sequential or combined. Earlier FECD stages might benefit from a sequential approach, as postoperative visual acuity might be good enough so that they can delay the need for a corneal transplant. Graft attachment and rebubbling rates might be better when using the sequential approach [**5**]. 

Visual outcomes for both sequential and combined approaches are similar at the 6-month mark [**6**]. In cases where anterior chamber contents are difficult to visualize due to central corneal edema or fibrosis, penetrating keratoplasty could be the preferred surgical approach. 

**Fig. 6:**
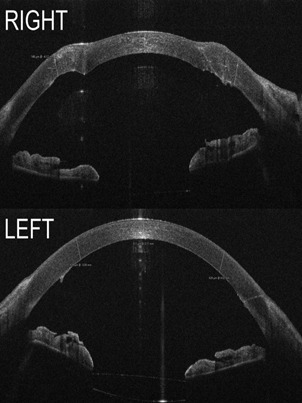
AS-OCT images. Above - Right eye, one year after penetrating keratoplasty, central corneal thickness 516 µm; Below - left eye, eight months after DMEK, central corneal thickness 516 µm (personal archive of Dr. Alina Gheorghe)

Understanding the biomechanics of the graft is key in postoperative management of refractive error post corneal transplantation to ensure the best visual recovery possible (**Fig. 6**). DMEK offers a more predictable refractive outcome. The most common refractive error after surgery is a hyperopic shift that must be considered when choosing the IOL [**7**]. PK management is more unpredictable, as corneal matrix remodeling and corneal shape vary and refraction is subject to change in the process of suture removal. Corneal topography is a valuable tool in assessing k values and planning suture removal. In our case, visual acuity was good in both eyes, however, the recovery of the left eye was faster, reaching very good visual acuity 1 month postoperatively. 

Graft rejection risk is significantly reduced in the case of posterior lamellar keratoplasty procedures compared to full-thickness keratoplasty [**8**].

DMEK is a very good treatment option for patients with Fuchs endothelial dystrophy, with excellent visual recovery and good graft survival at the 10-year mark [**9**,**10**].

## Conclusion

Corneal transplantation can nowadays offer the patient not only a chance for vision but also a very good recovery of visual acuity. This case showed, with ample documentation, the difference in duration of the recovery and the comparison of refractive error between penetrating keratoplasty and DMEK. While the visual recovery in both eyes at the one-year mark was very good, this case provided a unique opportunity to observe the real-world advantages and limits of penetrating keratoplasty and DMEK, as well as an opportunity to observe the challenges in the visual recovery process.

**Conflict of Interest Statement**

The authors state no conflict of interest.

**Informed Consent and Human and Animal Rights Statement**

Informed consent has been obtained from all individuals included in this study.

**Authorization for the use of human subjects**

Ethical approval: The research related to human use complies with all the relevant national regulations, and institutional policies, as per the tenets of the Helsinki Declaration, and has been approved by the review board of Clinical Hospital for Ophthalmological Emergencies Bucharest, Bucharest, Romania.

**Acknowledgments**

None.

**Sources of Funding**

None.

**Disclosures**

None.
